# Supplementary material for: Structure-function analysis of enterovirus protease 2A in complex with its essential host factor SETD3
Source: Nat Commun. 2022 Sep 8;13:5282. doi: 10.1038/s41467-022-32758-3 (PMC9453702; doi:10.1038/s41467-022-32758-3)
Supplement: Supplementary file 1 — Supplementary Information [file 41467_2022_32758_MOESM1_ESM.pdf]

Supplementary Information

for

Structure-function analysis of enterovirus protease 2A in complex with its essential host factor SETD3

Christine E. Peters[#], Ursula Schulze-Gahmen[#], Manon Eckhardt, Gwendolyn M. Jang, Jiewei Xu, Ernst H. Pulido, Conner Bardine, Charles S. Craik, Melanie Ott, Or Gozani, Kliment A. Verba*, Ruth Hüttenhain*, Jan E. Carette*, Nevan J. Krogan*[^]

[#] These authors contributed equally.
* These authors jointly supervised this work.
[^] Lead contact.

Department of Microbiology and Immunology, Stanford University School of Medicine, Stanford, CA, USA. (C.E.P., J.E.C.)
Gladstone Institute of Virology, The J. David Gladstone Institutes, San Francisco, CA, USA. (U.S.-G., M.O.)
Gladstone Institute of Data Science and Biotechnology, The J. David Gladstone Institutes, San Francisco, CA, USA. (M.E., E.H.P., N.J.K.)
Quantitative Biosciences Institute, University of California San Francisco, San Francisco, CA, USA. (M.E., G.M.J., J.X., E.H.P., C.B., C.S.C, M.O., K.A.V., R.H., N.J.K.)
QBI Coronavirus Research Group (QCRG), San Francisco, CA, USA (U.S.-G., M.E., G.M.J., E.H.P., C.B., C.S.C, M.O., K.A.V., R.H., N.J.K.)
Department of Cellular and Molecular Pharmacology, University of California, San Francisco, San Francisco, CA, USA (M.E., G.M.J., J.X., E.H.P., R.H., N.J.K.)
Department of Pharmaceutical Chemistry, University of California, San Francisco, San Francisco, CA, USA (C.B, C.S.C., K.A.V)
Department of Medicine, University of California, San Francisco, San Francisco, CA, USA (M.O.)
Chan-Zuckerberg Biohub, San Francisco, CA 94158, USA (M.O.)
Department of Biology, Stanford University, Stanford, CA, USA (O.G.)

Corresponding Author Emails
K.A.V.: Kliment.Verba@ucsf.edu, R.H.: Ruth.Huttenhain@ucsf.edu, J.E.C.: carette@stanford.edu, N.J.K.: nevan.krogan@ucsf.edu

Supplementary Figures

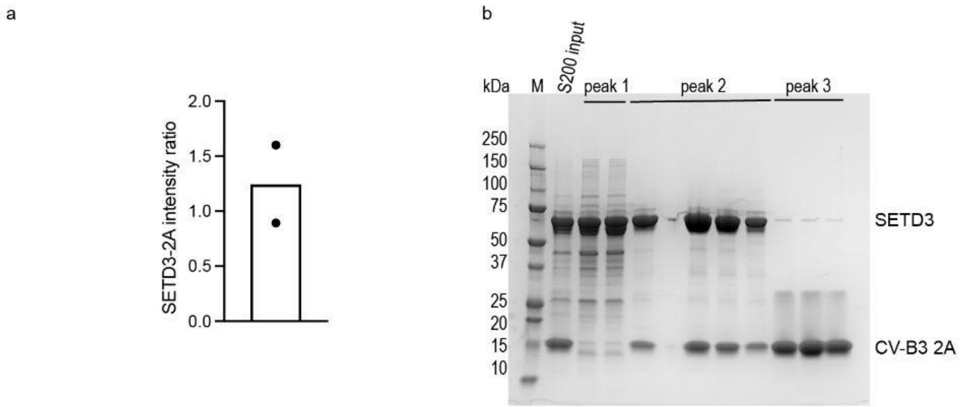

Supplementary Figure 1. SETD3-2A complex stoichiometry and reconstitution. Related to Fig. 1 and Fig. 2. (a) Stoichiometry of SETD3-2A complex. Absolute abundances for SETD3 and 2A were estimated from the sequential affinity purification MS data by summing up the top 3 peptide intensities per protein, assuming that the top 3 peptides per protein have a similar MS response. The ratio of estimated absolute abundances was calculated for SETD3 and 2A across two biological replicates. Source data are provided as a Source Data file. (b) Coomassie stained polyacrylamide gel of fractions eluted from Superdex 200 Increase size exclusion column after complex reconstitution. Molecular weight marker, column input, two fractions for peak 1 (aggregated material), four fractions for peak 2 (complex) and three fractions for peak 3 (monomeric 2A) were loaded on the gel. Lane 6 was skipped due to problems with the loading comb.

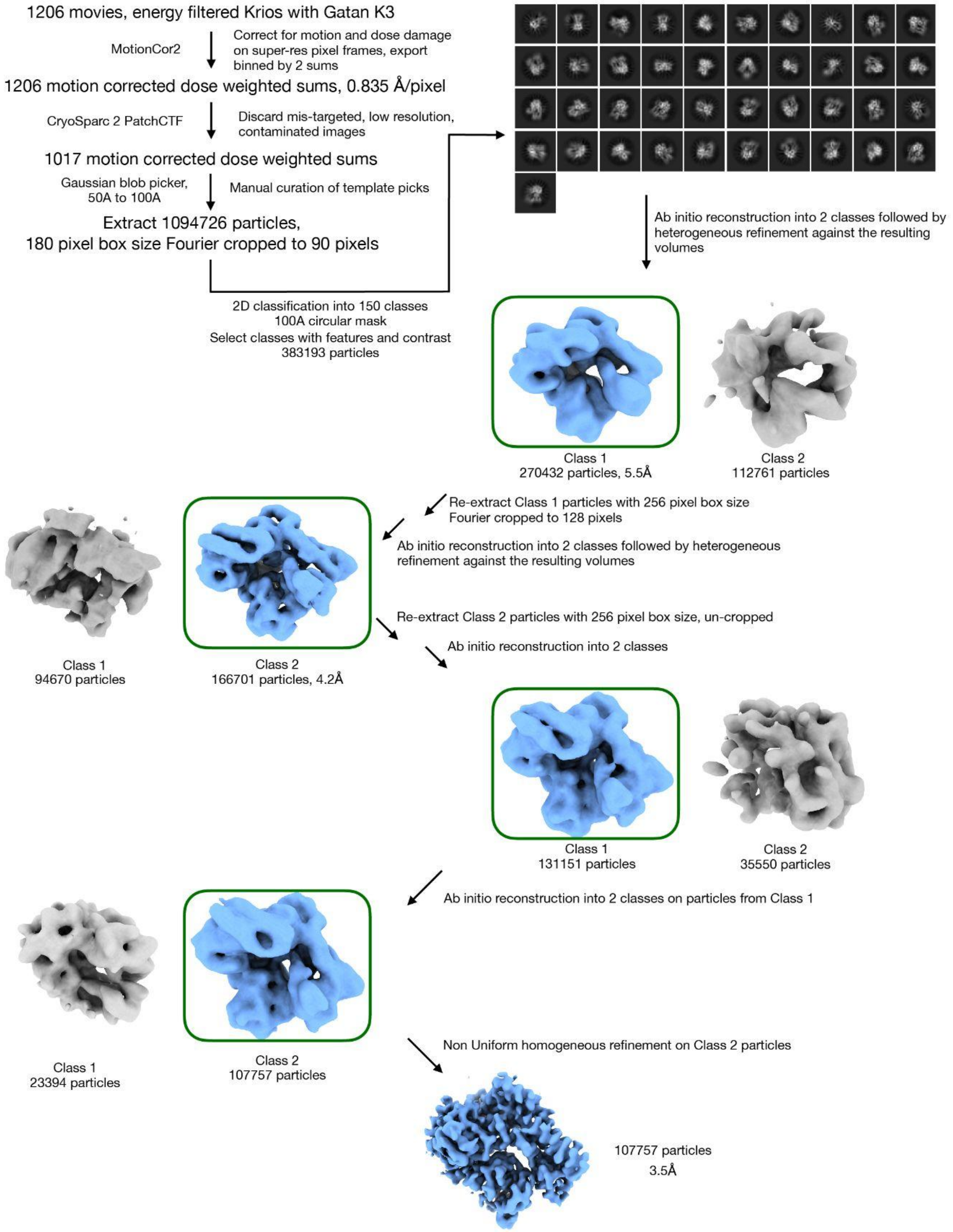

Supplementary Figure 2. Flowchart for SetD3-2A cryo-EM data collection and image processing. Related to Fig. 2. Detailed description of data collection parameters as well as image processing.

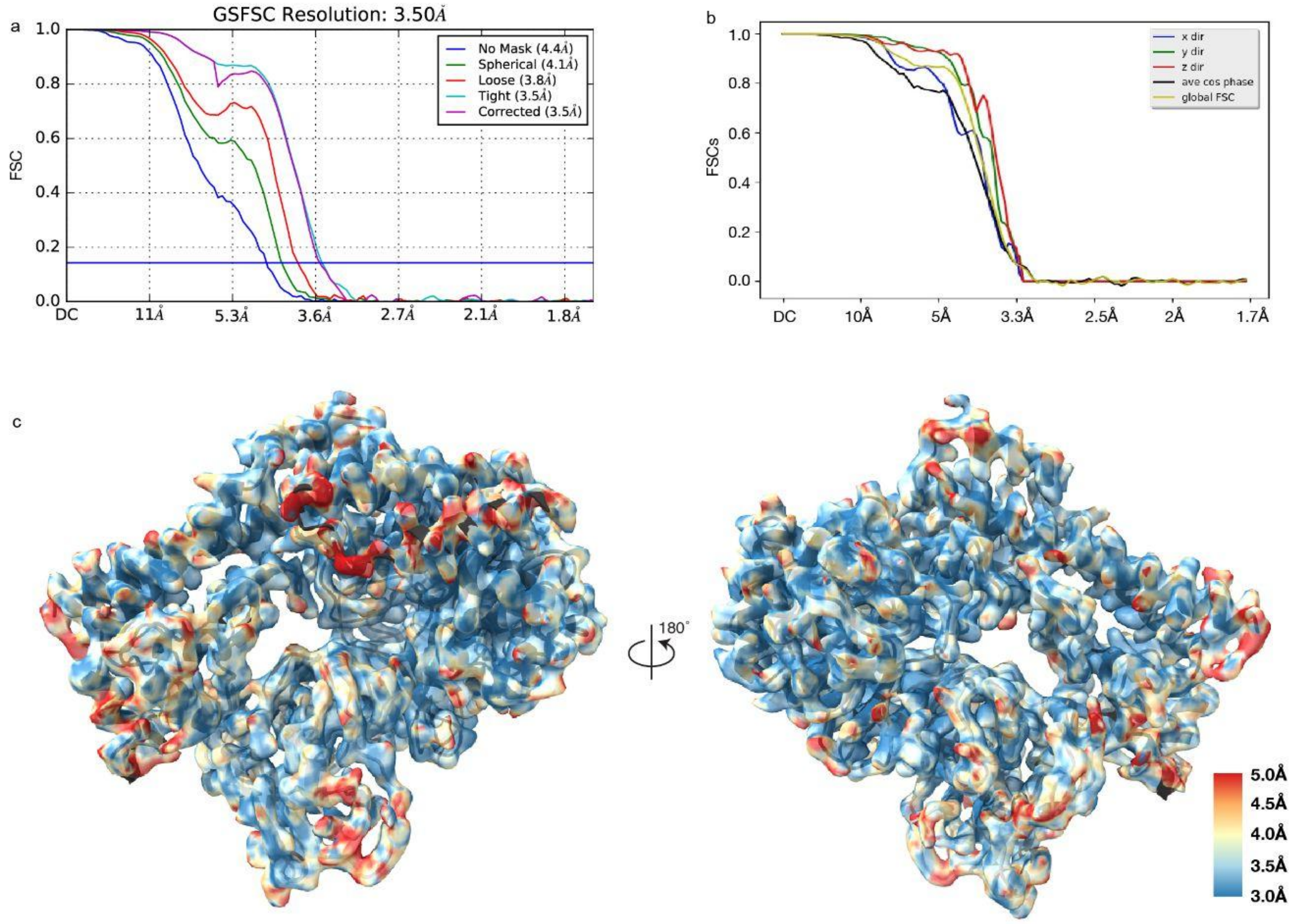

Supplementary Figure 3. Fourier shell correlation curves and local resolution estimation for the SETD3-2A cryo-EM reconstruction. Related to Fig. 2. (a) Gold standard Fourier shell correlation (FSC) curves for the last round of refinement out of cryoSPARC2 indicating a 3.5 Å cryo-EM reconstruction based on 0.143 FSC cut-off. (b) Output of the 3D FSC server plotting rotational FSCs for the reconstruction demonstrating isotropic resolution. Reported sphericity is 0.957. (c) Cryo-EM reconstruction of SETD3-2A protein complex colored by local resolution as reported by ResMap.

a

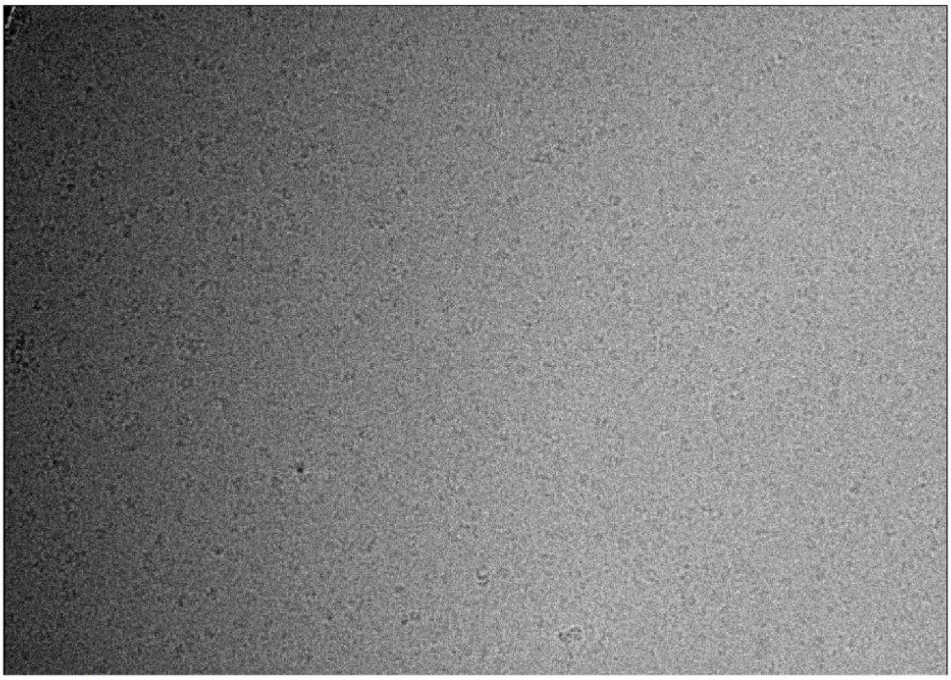

b

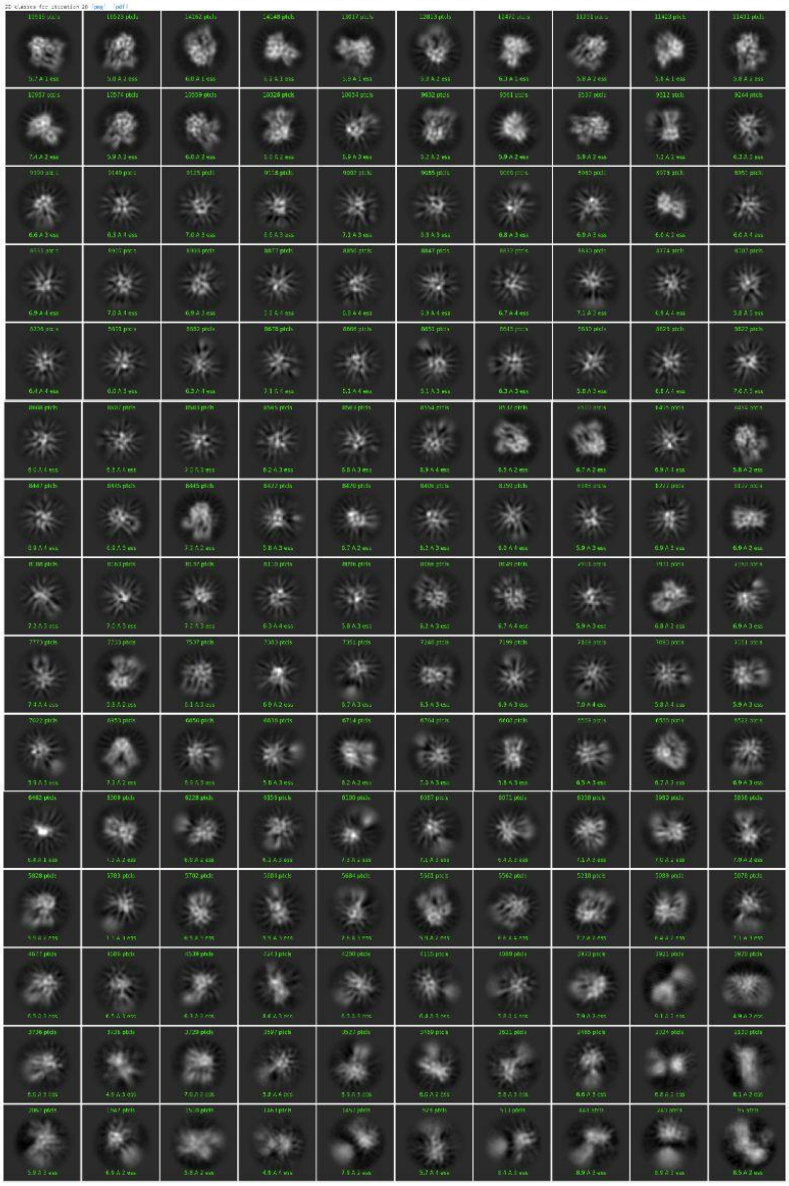

Supplementary Figure 4. Example micrograph and 2D classes. Related to Fig. 2. (a) Example micrograph of the SETD3-2A protein complex. (b) 2D classes during image processing.

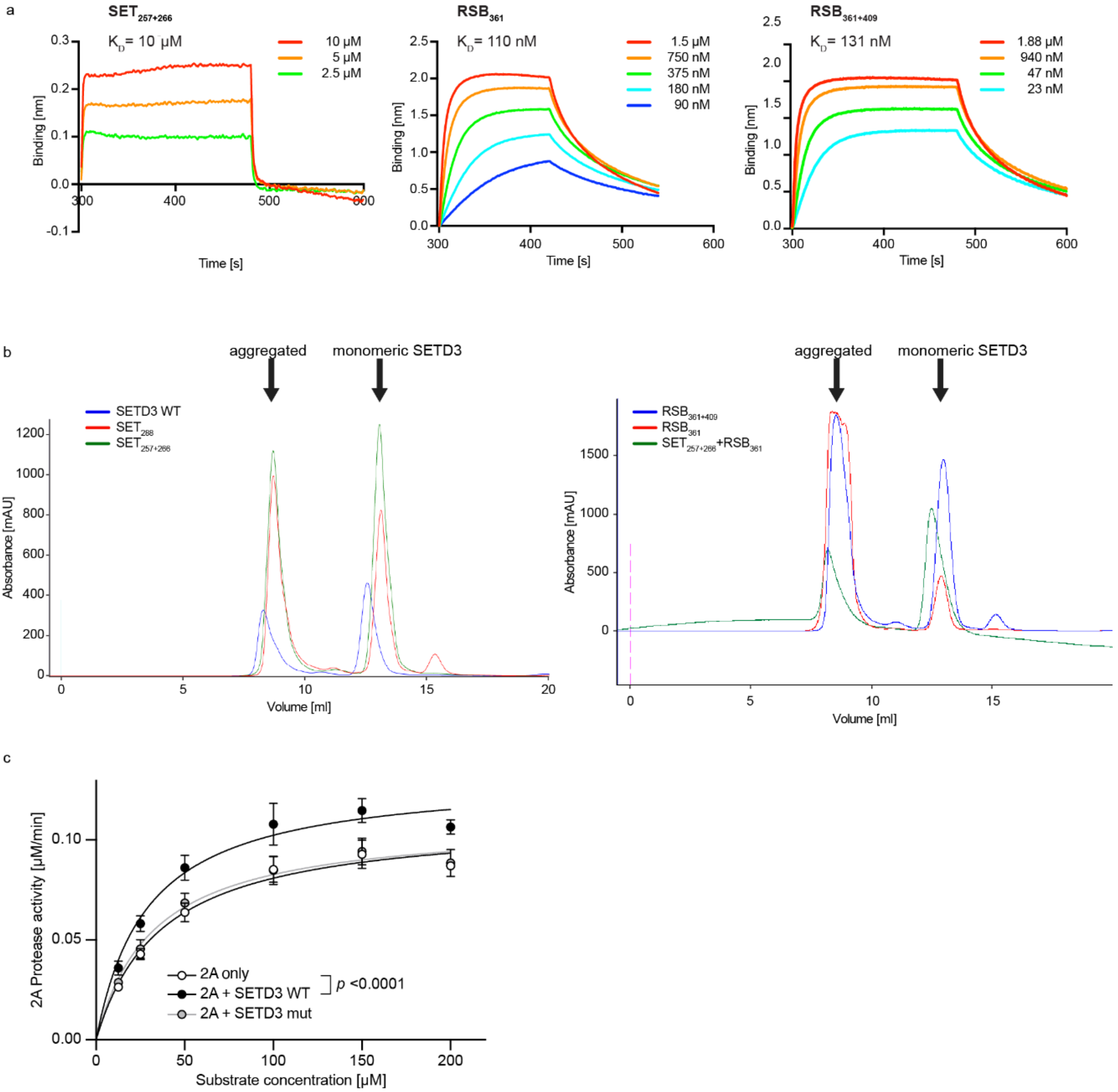

Supplementary Figure 5. Biolayer Interferometry and FRET-based protease assay. Related to Fig. 3. (a) BLI Results. Binding curves for varying input concentrations (different colors indicated in legend to individual plots) for selected SETD3 variants. Binding constants as calculated by the integrated software of the Octet Red384 instrument are shown. (b) SETD3 mutant protein purification. Elution profile from the final purification step on Superdex 200 Increase size exclusion column for SETD3 WT and mutants. Aggregated material and monomeric SETD3 peaks are indicated with arrows. (c) Michaelis Menten analysis of 2A protease activity. n=6 experiments at different peptide concentrations were performed in five replicates. The graph shows the mean values +/- standard deviation. Source data are provided as a Source Data file. Activities of 2A alone, and in complex with WT SETD3 and SETD3 triple mutant are compared. Statistical analysis using F-test to compare the curve fit under the assumption that K_m and k_{cat} are the same or different for 2A and 2A + SETD3 WT rejects the hypothesis that the variables are the same for the two data sets with P = 1.53263 x 10^{-13} and F (DFn, DFd) = 54.88 (2, 52). Curve fit and statistical analysis were performed using GraphPad Prism 9 (Version 9.3.1).

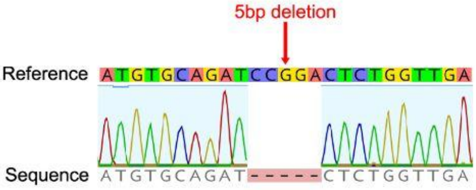

Supplementary Figure 6. Isogenic CRISPR-Cas9 deletion of SETD3 in H1-Hela^{+CDHR3} cells. Related to Fig. 4B and Fig. 5. Sequencing analysis of H1-Hela^{+CDHR3}#1C4 SETD3^{KO}#1B6 cells compared to parental H1-Hela^{+CDHR3}#1C4 cells (WT). Nucleotides 289-313 of SETD3 reference sequence are displayed.

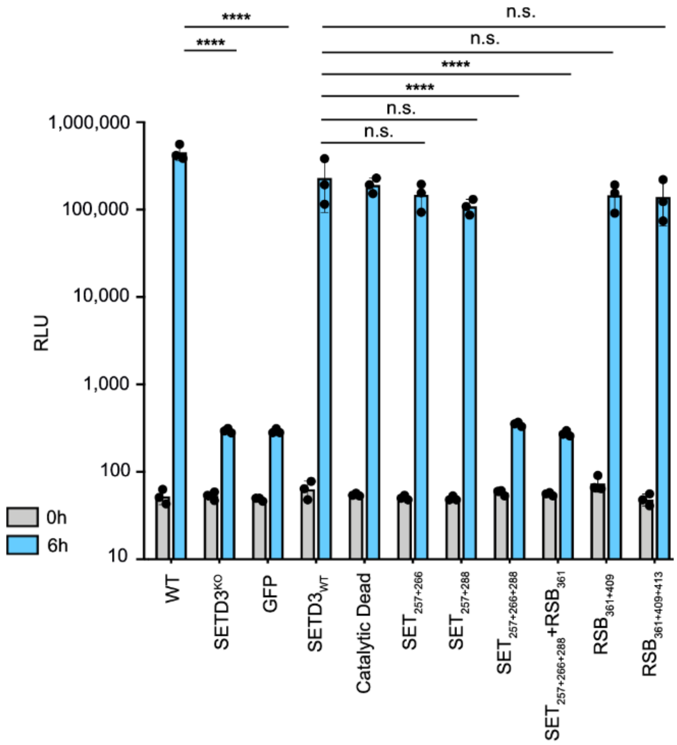

Supplementary Figure 7. SET interface mutants inhibit enterovirus infection upon overexpression. Related to Fig. 5. Infection of WT, $SETD3^{KO}$, or $SETD3^{KO}$ cells complemented with SETD3 structure derived mutants under a CMV promoter. n=3 biologically independent samples were measured for one experiment. Data are presented as mean and standard deviation. Source data are provided as a Source Data file. Statistics were performed on 6h timepoints using GraphPad Prism 9 (Version 9.3.1). *P*-values were determined by two-way ANOVA (Holm–Sidak corrected) on log-transformed data. RLU = relative light units, n.s. = not significant, $^{****}P \leq 0.0001$

**Data collection**	
Microscope	FEI Titan Krios
Voltage (keV)	300
Nominal Mag	105,000x
Exposure navigation	Stage position/beam and image shift
Cumulative dose (e/Å^2)	66
Requested defocus range (um)	1-2
Detector	Gatan K3
Detector Operation Mode	CDS
Pixel size (physical pixel, Å)	0.835
Dose rate (e^-/physical pixel/sec)	8
Total exposure time (sec)	6
Exposure per frame (sec)	0.05
Micrographs collected	1206
**Reconstruction**	EMD-23441
Initial particles used	1095000
Particles selected after 2D classification	383000
Particles used in final 3D reconstruction	108000
Symmetry Imposed	C1
Map Res (Å), masked/unmasked	3.5/4.4
FSC Threshold	0.143
Resolution range (local), Å	3-5
Final bfactor applied	-180
**Model Refinement**	PDB-7LMS
Initial Model (PDB)	4MG3, 6MBK
Protein residues (atoms)	612 (4920)
Ligands (atoms)	2 (27)
Map Correlation Coefficient (masked)	0.83
RMSD, Bond Lengths (Å)	0.019
RMSD, Bond Angles (°)	1.576
Ramachandran Outliers (%)	0
Ramachandran Allowed (%)	0.16
Ramachandran Favored (%)	99.84
MolProbity score	0.65
Clashscore (all atoms)	0.41
Rotamer outliers (%)	0

Supplementary Table 1. Data collection parameters and statistics on cryo-EM reconstruction and the SETD3-2A model. Related to Fig. 2

**SETD3 residue, # contact <4.1Å[a]**	**2A residues**	**Hydrogen bonds/salt bridges**
N256, 2	P70	
Q257, 10	**G58, V59, E114**	Q257 NE2 – E114 OE1
P259, 4	**H68**	
S264, 4	**R112**	
R265, 5	C47, D48	R265 NH2 – C47 O
V266, 12	**V59, R112, C113, E114**	
I284, 3	F101, P70	
T286, 3	**Y69,** P70	
G287, 2	**H68**	
Y288, 11	**K67, H68**	Y288 N – H68 O<br>Y288 O – H68 N
L290, 2	**H68**	
E291, 1	N66	
E296, 3	**K67**	E296 OE1 – K67 NZ
T315, 1	S72	
R336, 5	E74	R336 NH1 – E74 OE2<br>R336 NH2 – E74 OE2
A360, 1	S93	
G361, 5	**H94**	
A379, 1	L78	
Q380, 3	P76, G77	
F409, 12	N34, L78, Y91, S93	
G412, 6	L78, Y91	
N413, 10	Y91	

Supplementary Table 2. Intermolecular contacts between SETD3 and 2A. Related to Figs. 2 and 3. [a]Contacts were determined with the program ncont in the CCP4 program suite (Winn *et al*., 2011). Residues highlighted in bold were previously identified as critical for PPIs between SETD3 and 2A in a mammalian two-hybrid system (Diep *et al*., 2019).

Supplementary References

1. Winn, M. D. *et al.* Overview of the CCP4 suite and current developments. *Acta Crystallogr. D Biol. Crystallogr.* **67**, 235–242 (2011).
2. Diep, J. *et al.* Enterovirus pathogenesis requires the host methyltransferase SETD3. *Nat Microbiol* **4**, 2523–2537 (2019).
