# Supplementary material for: Structure-function analysis of enterovirus protease 2A in complex with its essential host factor SETD3
Source: Nat Commun. 2022 Sep 8;13:5282. doi: 10.1038/s41467-022-32758-3 (PMC9453702; doi:10.1038/s41467-022-32758-3)
Supplement: Supplementary file 2 — Description of Additional Supplementary Files [file 41467_2022_32758_MOESM2_ESM.pdf]

File name: Supplementary Data 1

Description: Separate Excel File. Mass spectrometry data of SETD3-FLAG APs. Related to Fig. 1.

File name: Supplementary Data 2

Description: Separate Excel File. Mass spectrometry data of SETD3-FLAG CV-B3-Strep double-APs. Related to Fig. 1.
