# Supplementary material for: Structure-function analysis of enterovirus protease 2A in complex with its essential host factor SETD3
Source: Nat Commun. 2022 Sep 8;13:5282. doi: 10.1038/s41467-022-32758-3 (PMC9453702; doi:10.1038/s41467-022-32758-3)
Supplement: Supplementary file 5 — Reporting Summary [file 41467_2022_32758_MOESM5_ESM.pdf]

nature portfolio

Corresponding author(s): Nevan Krogan

Last updated by author(s): Jul 29, 2022

Reporting Summary

Nature Portfolio wishes to improve the reproducibility of the work that we publish. This form provides structure for consistency and transparency in reporting. For further information on Nature Portfolio policies, see our Editorial Policies and the Editorial Policy Checklist.

Statistics

For all statistical analyses, confirm that the following items are present in the figure legend, table legend, main text, or Methods section.

n/a	Confirmed	
☐	☒	The exact sample size (*n*) for each experimental group/condition, given as a discrete number and unit of measurement
☐	☒	A statement on whether measurements were taken from distinct samples or whether the same sample was measured repeatedly
☐	☒	The statistical test(s) used AND whether they are one- or two-sided<br>*Only common tests should be described solely by name; describe more complex techniques in the Methods section.*
☒	☐	A description of all covariates tested
☐	☒	A description of any assumptions or corrections, such as tests of normality and adjustment for multiple comparisons
☐	☒	A full description of the statistical parameters including central tendency (e.g. means) or other basic estimates (e.g. regression coefficient) AND variation (e.g. standard deviation) or associated estimates of uncertainty (e.g. confidence intervals)
☐	☒	For null hypothesis testing, the test statistic (e.g. *F*, *t*, *r*) with confidence intervals, effect sizes, degrees of freedom and *P* value noted<br>*Give P values as exact values whenever suitable.*
☒	☐	For Bayesian analysis, information on the choice of priors and Markov chain Monte Carlo settings
☒	☐	For hierarchical and complex designs, identification of the appropriate level for tests and full reporting of outcomes
☒	☐	Estimates of effect sizes (e.g. Cohen's *d*, Pearson's *r*), indicating how they were calculated

Our web collection on statistics for biologists contains articles on many of the points above.

Software and code

Policy information about availability of computer code

Data collection	CryoEM data was collected with Serial EM 3.8.6 and Digital Micrograph 3.31.2359.0. Biolayer interferometry data acquisition was performed with Octet software version 10.0.1.6.
Data analysis	CryoEM data was analysed and processed using: SCI PION 2.0 (de la Rosa-Trevín et al., 2016), MotionCor2 vl.4.3, CTFFIND4 4.1.10 (Rohou and Grigorieff, 2015), cryoSPARC v2.15.0, ChimeraX 1.0, Rosetta 3.12, Coot 0.9.1, ISOLDE 1.0.1, Phenix 1.18.2., ResMap (Kucukelbir et al., 2014), the 3DFSC server (https://3dfsc.salk.edu/) (Tan et al., 2017), GraphPad Prism 8.4., and PyMOL 2.3.5. Homology modelling was performed using the SwissModel web-server [https://swissmodel.expasy.org/]. Structure analysis was performed using the protein interfaces, surfaces and assemblies service PISA at the European Bioinformatics Institute (http://www.ebi.ac.uk/pdbe/prot_int/pistart.html) (Krissinel & Henrick, 2007) as well as NCONT in CCP4i 7.1 (Winn et al, 2011). MS data was analysed using MaxQuant data analysis algorithm (version 1.5.2.8 and version 1.6.12.0) (Cox and Mann, 2008) as well as MSstats (version 4.2.0) and SAINTexpress (Choi et al., 2011, Choi, et al., 2012). Biolayer interferometry data analysis was performed with Octet software version 10.0.1.6. GraphPad Prism 9 software (version 9.3.1) was used for data visualisation, curve fitting and statistical analysis.

For manuscripts utilizing custom algorithms or software that are central to the research but not yet described in published literature, software must be made available to editors and reviewers. We strongly encourage code deposition in a community repository (e.g. GitHub). See the Nature Portfolio guidelines for submitting code & software for further information.

Data

Policy information about availability of data

All manuscripts must include a data availability statement. This statement should provide the following information, where applicable:

- Accession codes, unique identifiers, or web links for publicly available datasets
- A description of any restrictions on data availability
- For clinical datasets or third party data, please ensure that the statement adheres to our policy

The mass spectrometry-based proteomics data have been deposited to the ProteomeXchange Consortium via the PRIDE (Perez-Riverol et al., 2019) partner repository with the dataset identifier PXD024127 [https://www.ebi.ac.uk/pride/archive/projects/PXD024127]. MS data was searched against the SwissProt Human protein sequences [https://ftp.uniprot.org/pub/databases/uniprot/previous_releases/release-2018_07/knowledgebase/]. The accession numbers for the cryo-EM structure of CV-B3 2A bound to SETD3 reported in this paper are PDB:7LMS and EMDB:23441. Our structure was fit with a homology model for the 2A protease based on PDB:4MG3 [http://doi.org/10.2210/pdb4MG3/pdb], and the existing SETD3 model (PDB:6MBK Chain A [http://doi.org/10.2210/pdb6MBK/pdb]). Source data for all figures are provided with this paper.

Human research participants

Policy information about studies involving human research participants and Sex and Gender in Research.

Reporting on sex and gender	N/A
Population characteristics	N/A
Recruitment	N/A
Ethics oversight	N/A

Note that full information on the approval of the study protocol must also be provided in the manuscript.

Field-specific reporting

Please select the one below that is the best fit for your research. If you are not sure, read the appropriate sections before making your selection.

☒ Life sciences ☐ Behavioural & social sciences ☐ Ecological, evolutionary & environmental sciences

For a reference copy of the document with all sections, see nature.com/documents/nr-reporting-summary-flat.pdf

Life sciences study design

All studies must disclose on these points even when the disclosure is negative.

Sample size	No sample-size calculations were performed for this study. The number of required independent biological replicates that were performed was determined, as common in the field, by the requirement for statistical significance and reproducibility. It is an accepted practice in the field of proteomics, that biological triplicate measurements of AP-MS samples are sufficient for measuring high confidence interactions using the methods and software performed in this study. Three biological replicates were independently prepared for affinity purification. All viral infection experiments were performed in biological triplicate measurements. CETSA experiments were conducted across three independent experiments. Six experiments at different peptide concentrations were performed in five replicates for protease activity experiments. For bilayer interferometry binding assays, conditions were optimised over 2-3 repeated experiments. Kinetic data were obtained from one optimised experiment which includes binding curves at a range of various concentrations for one of the binding partners. Inhibition BLI assays were conducted at a fixed protein concentration in triplicate independent experiments to allow for statistical analysis.
Data exclusions	No data were excluded from analyses.
Replication	All attempts at replication were succesful. The number of biologically independent replicates are indicated in the figure legends.
Randomization	Sample randomization is not relevant to our study because experimental groups do not exist.
Blinding	Blinding is not relevant to the AP-MS data because our data are acquired and processed systematically with established scoring algorithms, excluding human bias. Blinding was not performed for all other experiments, as knowledge about the treatment conditions was required for the researchers to be able to perform the experiments and ensure proper sample handling.

Reporting for specific materials, systems and methods

We require information from authors about some types of materials, experimental systems and methods used in many studies. Here, indicate whether each material, system or method listed is relevant to your study. If you are not sure if a list item applies to your research, read the appropriate section before selecting a response.

Materials & experimental systems

n/a	Involved in the study
☐	☒ Antibodies
☐	☒ Eukaryotic cell lines
☒	☐ Palaeontology and archaeology
☒	☐ Animals and other organisms
☒	☐ Clinical data
☒	☐ Dual use research of concern

Methods

n/a	Involved in the study
☒	☐ ChIP-seq
☒	☐ Flow cytometry
☒	☐ MRI-based neuroimaging

Antibodies

Antibodies used

SETD3 (1:5,000 western blot; Abcam, ab176582;) Actin (1:2,000 western blot; Sigma-Aldrich, A2066), FLAG M2 (1:2,000 western blot; Sigma–Aldrich, F3165), H73(3-me) (1:1,000 western blot; (Wilkinson et al., 2019)), GAPDH (1:5,000 western blot; GeneTex, GTX627408) and mouse HRP secondary (1:10,000; GeneTex, GTX213111-01) or rabbit HRP secondary (1:10,000; GeneTex, GTX213110-01). Alterntatively, primary antibodies directly conjugated to HRP were used Strep HRP (1:10,000 western blot; Sigma-Aldrich, 71591), Streptactin HRP (1:500 western blot; BioRad, 1610381) and FLAG M2 HRP (1:5000 western blot; Sigma-Aldrich, A8592).

Validation

Flag M2 antibody was validated by the vendor: it detects a single band of protein on a Western Blot from mammalian crude cell lysates. Strep tag II antibody was validated by the vendor: it displays negligible cross-reactivity with bacterial, mammalian, and insect lysates. SETD3 antibody was validated by loss of detection after CRISPR/Cas9 knockout with multiple guide RNAs. Actin, FLAG M2, GAPDH, Streptactin-HRP and secondary antibodies were authenticated by the vendor. Anti-actin-H73(3-me) polyclonal antibody was validated by western blotting with cell extracts where actin methylation was quantified by mass spectrometry.

Eukaryotic cell lines

Policy information about cell lines and Sex and Gender in Research

Cell line source(s)

HEK293T/17 (ATCC CRL-11268), H1-Hela (ATCC CRL-1958), 293FT (Fisher Scientific R70007). Information on cell line generation can be found in the Methods section "Lentiviral packaging" and "Generation of H1Hela+CDHR3 isogenic CRISPR–Cas9 SETD3 KO cell line."

Authentication

The cells were purchased and routinely maintained in our lab. They were not authenticated experimentally for these studies. For generated cell lines, expression of proteins of interest were validated by western blot.

Mycoplasma contamination

Cell lines used for viral infection assays, actin methylation WB and CETSA experiments were tested negative for mycoplasma using the MycoAlertTM PLUS Mycoplasma Detection Kit (Lonza, Cat#LT07-710). Cells used for AP-MS, AP-WB and double-IP experiments were not tested for mycoplasma contamination.

Commonly misidentified lines
(See ICLAC register)

No commonly misidentified cell lines were used in this study.
